# Investigating the Sexual Dimorphism of Waist-to-Hip Ratio and Its Associations with Complex Traits

**DOI:** 10.3390/genes16060711

**Published:** 2025-06-16

**Authors:** Haochang Li, Shirong Hui, Xuehong Cai, Ran He, Meijie Yu, Yihao Li, Rongbin Yu, Peng Huang

**Affiliations:** Department of Epidemiology, Center for Global Health, School of Public Health, Key Laboratory of Public Health Safety and Emergency Prevention and Control Technology of Higher Education Institutions in Jiangsu Province, National Vaccine Innovation Platform, Nanjing Medical University, Nanjing 211166, China; lihaochang@stu.njmu.edu.cn (H.L.); huishirong@stu.njmu.edu.cn (S.H.); caixuehong@stu.njmu.edu.cn (X.C.); ranyhe@stu.njmu.edu.cn (R.H.); yumeijie1016@stu.njmu.edu.cn (M.Y.); liyihao0915@stu.njmu.edu.cn (Y.L.); rongbinyu@njmu.edu.cn (R.Y.)

**Keywords:** WHR, sexual dimorphism, genetic architecture, genetic correlation, PGS, sex-specific traits

## Abstract

**Background:** Obesity significantly impacts disease burden, with waist-to-hip ratio (WHR) as a key obesity indicator, but the genetic and biological pathways underlying WHR, particularly its sex-specific differences, remain poorly understood. **Methods:** This study explored WHR’s sexual dimorphism and its links to complex traits using cross-sectional surveys and genetic data from Giant and UK Biobank (UKB). We analyzed WHR heritability, performed tissue-specific transcriptome-wide association studies (TWAS) using FUSION, and conducted genetic correlation analyses with linkage disequilibrium score regression (LDSC) and Local Analysis of [co]Variant Association (LAVA). Polygenic scores (PGS) for WHR were constructed using the clumping and thresholding method (CT), and associations with complex traits were assessed via logistic or linear models. **Results:** The genetic analysis showed sex-specific heritability for WHR, with TWAS identifying female-specific (e.g., *CCDC92*) and male-specific (e.g., *UQCC1*) genes. Global genetic correlation analysis revealed sex-specific associations between WHR and 23 traits, while local analysis identified eight sex-specific loci across five diseases. Regression analysis highlighted sex-specific associations for 70 traits with WHR and 45 traits with WHR PGS, with stronger effects in females. Predictive models also performed better in females. **Conclusions:** This study underscores WHR’s sexual dimorphism and its distinct associations with complex traits, offering insights into sex-specific biological differences, health management, and clinical advancements.

## 1. Introduction

Obesity is a chronic metabolic disease caused by excessive accumulation of body fat [1]. The prevalence of overweight and obesity is increasing [2], with more than 1 billion people estimated to be obese worldwide [3,4]. This staggering prevalence underscores the urgent need to better understand the complex biological mechanisms underlying excessive fat accumulation and its associated health risks. Indicators such as body mass index (BMI) and waist-to-hip ratio (WHR) are widely used to evaluate the degree of obesity [5,6] and its associated health risks [7,8,9]. While BMI is widely used to assess obesity, it may overestimate risks in certain populations due to racial differences in body composition [10]. In contrast, WHR, which considers the distribution of body fat and is closely linked to visceral or abdominal fat, provides a more precise measure of obesity-related disease risks [11]. However, while the mechanisms linking BMI to obesity are well-established, the genetic and biological pathways underlying WHR, particularly its sex-specific differences, remain poorly understood.

A particularly intriguing aspect of WHR is its pronounced sexual dimorphism. Men and women exhibit fundamentally different fat distribution patterns that are evident from puberty onward and persist throughout adulthood [12,13,14]. Specifically, women typically exhibit higher gluteofemoral fat while men show greater visceral fat. Genome wide association studies (GWASs) have identified multiple loci associated with WHR, some of which exhibit significant sex-specific effects [15,16,17,18]. For instance, certain genetic variants (e.g., *TACR1*, *IGFL2*, *CNTN5*, and *GPC6*) demonstrate stronger effects in women [15,17], suggesting distinct genetic architectures between sexes. These findings suggest that the genetic architecture of fat distribution differs meaningfully between sexes, potentially reflecting differential regulation by sex hormones or other biological factors [19]. Another Mendelian randomization analysis revealed the differences in the association between WHR and mortality between men and women under different sexes [20]. However, most genetic analyses treat sex as a confounder rather than exploring its role in shaping WHR-associated genetic risk, leaving a critical gap in our understanding of obesity biology.

Thus, we hypothesize that WHR-associated genetic variants exert differential effects between sexes, contributing to distinct fat distribution patterns and disease risks. Understanding these sex-specific genetic effects has important implications for both clinical practice and biomedical research. From a clinical perspective, recognition of sex-differential genetic risk factors could enable more personalized approaches to obesity prevention and treatment. From a research standpoint, identifying sex-specific WHR loci could reveal novel biological pathways involved in adipose tissue biology and provide new targets for pharmacological intervention.

To test this, we integrated WHR GWAS summary statistics with expression quantitative trait loci (eQTL) data to identify sex-specific genetic mechanisms. Moreover, we further assessed its genetic correlations with 353 complex traits and evaluated the predictive power of WHR and its polygenic score (PGS) across sexes. We aimed to provide new insights that could ultimately lead to more effective, sex-specific strategies for preventing and treating obesity and its associated disorders.

**Figure 1:**
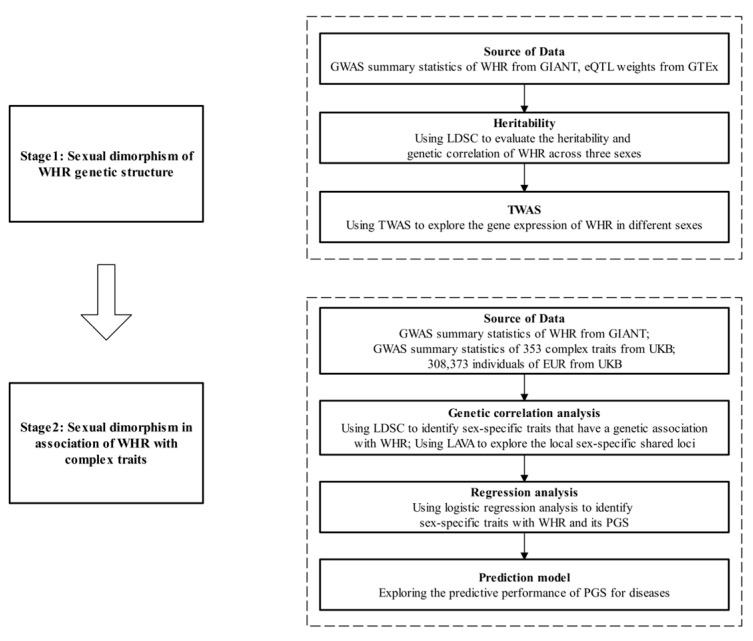
The flowchart of this study. We performed two stages of research. In the first stage, we mainly explored the sex dimorphism of the WHR genetic structure. Specifically, we used LDSC to evaluate the heritability and genetic correlation of WHR across three sexes and integrated WHR GWAS summary statistics with eQTL data to explore the gene expression of WHR in different sexes using TWAS. In the second stage, we concentrated on sexual dimorphism in the association of WHR with 353 complex traits. Specifically, we firstly integrated GWAS summary statistics of WHR and complex traits, respectively using LDSC to identify sex-specific traits that have a genetic association with WHR and LAVA to explore the local sex-specific shared loci. Secondly, we performed logistic regression analysis to identify sex-specific traits with WHR and its PGS in 308,373 individuals of EUR from UKB, respectively. Finally, we explored the performance of WHR PGS for diseases in prediction models.

The flowchart of this study can be seen at Figure 1.

## 2. Methods

### 2.1. GWAS Summary Statistics of WHR

We downloaded three GWAS summary statistics for WHR in the European (EUR) population measured from the GIANT consortium [18]. Specifically, we included female-specific summary statistics concerning 118,004 individuals, male-specific summary statistics concerning 94,434 individuals, and the mixed summary statistics. Following the quality control measures [21], we removed single nucleotide polymorphisms (SNPs): (i) duplicated, (ii) with minor allele frequency (MAF) < 0.01, and (iii) excluded from 1000 GP. Finally, we obtained 2,404,101 SNPs for mixed data, 2,387,331 SNPs for female data, and 2,166,105 SNPs for male data.

### 2.2. Study Population

UK Biobank (UKB) contains physical measurement indicators, socioeconomic and lifestyle factors, health and medical history, and genetic variants information of approximately 500,000 participants enrolled between 2006 and 2010 [22]. This study used the data of participants from enrollment through 14 September 2024. Our study protocol has been approved by all necessary committees of the UKB (Application Number: 144904).

Besides gender and age, we extracted anthropometric variables, including waist circumference, hip circumference, and BMI, and lifestyle factors including smoking, drinking, sleep duration, time spent in light physical activity per week, and time spent in vigorous physical activity per week. We defined physical activity as the sum of time spent in light and vigorous physical activity per week [23]. Notably, we used baseline survey data to define the status of physical measurements and lifestyle factors for each participant. Following previous studies [24,25,26], we retained individuals: (i) with European ancestry; (ii) included in the genotype principal component calculation; (iii) whose self-reported sex matched the genetic sex; (iv) with specific waist circumference and hip circumference measurement data; and (v) without missing information on lifestyle factors or other covariates. After quality control and exclusion of missing values, we retained 308,373 individuals, including 165,607 females and 142,766 males.

### 2.3. Transcriptome-Wide Association Analysis

We performed tissue-specific transcriptome-wide association analysis (TWAS) using FUSION (version 3) [27] to investigate the specificity of gene expression of WHR across different sexes. FUSION estimates significant cis-genetic effects on SNPs within 500 kb of gene boundaries using various penalized linear models (GBLUP, LASSO, Elastic Net, etc.) to build prediction models, and then selects the best model based on the prediction results [27,28]. In this study, we downloaded the weight files for whole blood from GTEx V8 and performed TWAS using the default options in FUSION. The significant genes were defined as those with Bonferroni-adjusted *p* value < 0.05.

### 2.4. GWAS Summary Statistics for Complex Traits

As for UKB phenotypes, we retrieved GWAS summary statistics related to diseases, anthropometric, blood cell counts, and biochemical markers from the Neale Lab (http://www.nealelab.is/uk-biobank (accessed on 25 September 2024)). For diseases, we excluded the infectious diseases, neonatal related diseases, and congenital malformations [29]. For anthropometric, we excluded measurement of waist circumference, hip circumference, weight, and height. For blood cell counts, we selected the categories “Biological samples > Blood assays > Blood count”. For biochemical markers, we selected the categories “Biological samples > Blood assays > Blood biochemistry” and “Biological samples > Blood assays > Neurobiomarkers”. All of the GWAS summary statistics we selected covered male, female, and mixed sexes. Finally, we obtained 261 summary statistics associated with diseases, 92 summary statistics associated with measurement traits including 31 associated with body measurements, 31 associated with blood cell counts, and 30 associated with biochemical markers (Table S1).

### 2.5. Genetic Correlation Analysis

We used linkage disequilibrium score regression (LDSC) (version 1.0.1) [30] to (i) calculate the linkage disequilibrium (LD) scores of the reference panel across different sexes, (ii) estimate the heritability of WHR, (iii) estimate the global genetic correlation (rg) of WHR across different sexes, and (iv) estimate the global genetic correlation (rg) between WHR and UKB phenotypes across different sexes. We used 1000 Genomes Project Phase 3 European population as the reference panel [31]. The window for LD scores was set to 1 Mb, while nominal global genetic correlations were defined as rg > 0 and *p* value < 0.05 and significant global genetic correlations were defined as rg > 0 and a Bonferroni-adjusted *p* value < 0.05 (adjusted *p* < 1.42 × 10^−4^, 0.05/353).

We used Local Analysis of [co]Variant Association (LAVA) (version 1.0.0) [32] to perform local genetic correlation analysis to detect the local shared genetic basis of WHR with diseases in the female and male sets. We downloaded information roughly equal to the size of the semi-independent genome (https://github.com/cadeleeuw/lava-partitioning (accessed on 16 December 2024)). In brief, the whole genome was divided into 2495 semi-independent LD blocks, using the default values of the block algorithm and a minimum block size of 2500 base pairs [32,33,34]. We used the *run.univ.bivar* function to perform univariate and bivariate tests in sequence. The *p*-value threshold for the univariate test was set as 0.05/2495. Then we performed bivariate tests for the phenotypes that reached the univariate threshold. The significant bivariate loci were defined as those with a Bonferroni-adjusted *p* value < 0.05, and nominal results were defined as *p* value < 0.05.

### 2.6. Polygenic Risk Score

We employed the clumping and thresholding method (CT) to estimate the PGS of WHR [35]. For mixed summary statistics, we used 50,000 and 258,373 individuals as the validation set and test set, respectively. We used the validation set to select the best parameter combinations and the test set to evaluate the prediction performance. Adjusting for sex, age, and the top ten genetic principal components (PCs), we constructed a PGS for individuals in the test set. Following [36], we selected three hyper-parameters from among 2800 possible combinations, which are drawn from 50 *p*-value thresholds, four window sizes (50, 100, 200 and 500 kb), and seven *R*^2^ values (0.01, 0.05, 0.1, 0.2, 0.5, 0.8 and 0.9). We used the R package *bigsnpr* (version: 1.12.2) [37] to fit the model. We used 503 EUR in 1000 GP as the reference panel. We constructed the PGS in the test set using PLINK1.9 (version 1.0.0).

In addition, we applied the same strategy in the two sex-specific PGS summary statistics. We used 26,914 females and 23,086 males to construct the sex-specific validation set. Adjusting for age and the top ten PCs, we constructed the PGS for 138,693 females and 119,680 males in the test set.

### 2.7. Definition of Outcome

We defined two categories of outcomes including diseases and measurement traits (including body measurements, blood cell counts, and biochemical markers). In detail, we selected the phenotypes with nominal global genetic correlation with WHR in mixed sexes. For the outcomes of measurement traits, we used baseline data. Finally, we obtained 90 outcomes for diseases and 57 outcomes for measurement traits.

### 2.8. Statistical Analysis

First, for baseline characteristics, we used the mean ± standard deviation (SD) to describe continuous variables, and numbers and percentages to describe categorical variables. For the differences between the sexes, we used the unpaired, 2-tailed *t* test to compare the continuous variables, and the χ^2^ test for categorical variables. Second, we used multivariable logistic regression to test the association of WHR with the diseases category and multiple linear regression to test the association of WHR with measurement traits. For the mixed-sex population, we adjusted the covariates of sex, base age, BMI, Townsend deprivation index, smoking status, sleeping duration, physical activity, and alcohol drinking status; for sex-specific populations, we did not adjust for sex compared with the mixed-sex population. Third, we used a multivariable logistic regression model to test the association of PGS with diseases and a multiple linear regression to test the association of PGS with measurement traits. For the mixed-sex population, we adjusted the covariates of sex, age, top ten principal components (PCs), and BMI, and for the sex-specific populations, we treated age, top ten PCs, and BMI as covariates. Finally, we calculated the area under the curve (AUC) value to evaluate the predictive ability of the logistic regression models and the R^2^ to evaluate the fitting precision of the linear regression models that included WHR PGS. To ensure the rigor of the results, we applied false discovery rate (FDR), the Bonferroni correction was applied and *p* < 4.30 × 10^−4^ (0.05/147) was considered statistically significant.

## 3. Results

### 3.1. Sexual Dimorphism from Association Study

**Figure 2:**
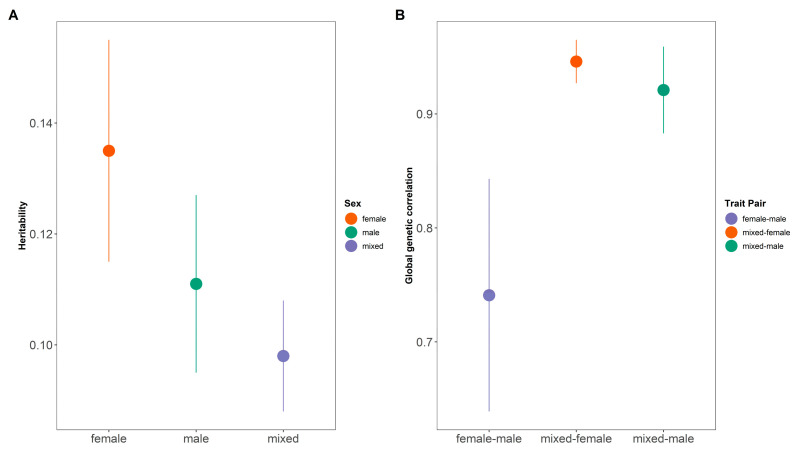
**The heritability and global genetic correlation of WHR across three sexes.** (**A**) The heritability of WHR of three sexes. The heritability of females is greater than that of males, followed by the mixed sexes. The *x*-axis represents different genders while the *y*-axis represents heritability. (**B**) The global genetic correlation of WHR across three sexes. The genetic correlation between females and mixed sexes is stronger than that between males and mixed sexes. The *x*-axis represents different gender pairs while the *y*-axis represents global genetic correlation. WHR, waist-to-hip ratio.

Based on LDSC, the heritability for males (h^2^ = 0.111, 95% confidential interval [CI]: 0.095–0.127) was significantly higher than that for mixed sexes (h^2^ = 0.098, 95% CI: 0.088–0.108). The difference of heritability for females (h^2^ = 0.135, 95%: 0.115–0.155) is not significant to that for males and mixed sexes (Figure 2A). The genetic correlation between females and males (rg = 0.741, 95% CI: 0.639–0.843) was significantly lower than that between females and mixed sexes (rg = 0.946, 95% CI: 0.927–0.965) and that between males and mixed sexes (rg = 0.921, 95% CI: 0.883–0.959) (Figure 2B).

**Figure 3:**
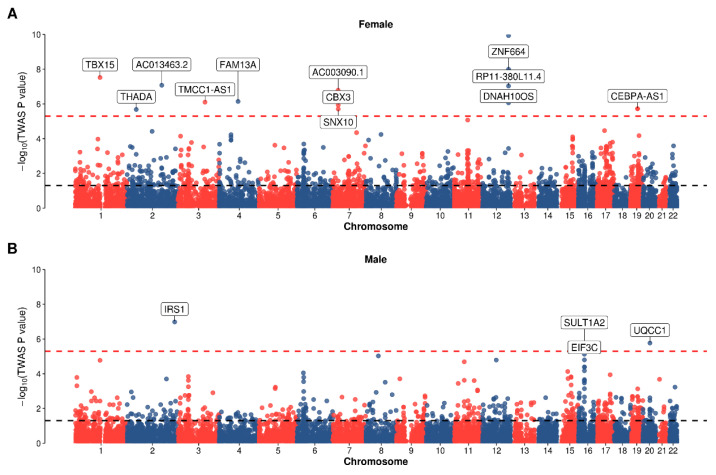
**TWAS results of WHR in adipose subcutaneous tissue.** (**A**) TWAS results of WHR in the female set; (**B**) TWAS results of WHR in the male set. In the adipose subcutaneous tissue, 12 genes showed strong associations with females, while four genes showed strong associations with males. The signals of related genes in females were stronger than those in males. The *x*-axis represents the chromosome number while the *y*-axis represents the −log_10_ of the TWAS *p* value. Each dot represents a gene. The solid red horizontal line is marked at the Bonferroni threshold of significance for multiple testing, and the black dotted horizontal line represents the *p* < 0.05. TWAS, transcriptome-wide association analysis; WHR, waist-to-hip ratio.

Then, we used TWAS to detect the associated gene of WHR among adipose subcutaneous, adipose visceral omentum, and whole blood across female and male categories (Figure 3, Table S2, Figures S1 and S2). We defined 2489 nominal genes and 31 sex-specific genes after a Bonferroni correction. For WHR for females, FUSION defined 1548 nominal genes (*p* < 0.05) and 21 significant genes (Bonferroni-adjusted *p* value < 0.05), such as *RIMKLBP2* in adipose subcutaneous (Z = 8.595, *P*_adjusted = 8.10 × 10^−14^), *TBX15* in adipose visceral omentum (Z = 6.167, *P*_adjusted = 5.41 × 10^−6^), and *PLXND1* in whole blood (Z = 6.309, *P*_adjusted = 2.23 × 10^−6^). For WHR for males, FUSION defined 1171 nominal genes and 10 significant genes, such as *IRS1* in adipose subcutaneous (Z = 5.320, *P*_adjusted = 9.68 × 10^−4^), *UQCC1* in adipose visceral omentum (Z = 4.838, *P*_adjusted = 0.010), and *SULT1A2* in whole blood (Z = 4.848, *P*_adjusted = 0.009). Specifically, we defined two kinds of sex-specific genes: (i) male-specific genes with significantly larger effect size in males than that in females; (ii) female-specific genes with significantly larger effect size in females than that in males. For example, *CCDC92* is one of the female-specific genes with association in adipose subcutaneous (female: *p* = 1.17 × 10^−10^, male: *p* = 0.179), adipose visceral omentum (female: *p* = 9.71 × 10^−10^, male: *p* = 0.173), and whole blood (female: *p* = 2.15 × 10^−6^, male: *p* = 0.299). However, *UQCC1* is one of the male-specific genes with association in adipose subcutaneous (male: *p* = 1.70 × 10^−6^, female: *p* = 0.066), adipose visceral omentum (male: *p* = 1.31 × 10^−6^, female: *p* = 0.056), and whole blood (male: *p* = 2.00 × 10^−6^, female: *p* = 0.058).

In total, the results of heritability and TWAS indicated the sexual dimorphism of WHR.

### 3.2. Sexual Dimorphism for WHR with 147 Complex Traits

Based on LDSC, there remained 147 complex traits with nominal genetic correlations with WHR in the mixed-sex set (*p* < 0.05), including 90 diseases (e.g., chronic ischemic heart disease, rg = 0.486, *p* = 3.04 × 10^−27^) and 57 measurement traits (e.g., leg fat percentage (left), rg = 0.984, *p* = 3.06 × 10^−74^) (Table S3). Using genetic correlation analysis, we defined 43 significant traits for males, consisting of two diseases and 41 measurement traits (Table S4), and 51 significant traits for females, including five diseases and 49 measurement traits (Table S5), with WHR using a Bonferroni correction. Among them, two diseases (chronic ischemic heart disease and gonarthrosis [arthrosis of knee]) and 39 measurement traits were both correlated to WHR with a significant coefficient between the two sexes. However, three diseases and eight measurement traits were only significant in either the female-specific or male-specific set. For the male-specific set, we detected monocyte count (male: rg = 0.278, 95%CI = 0.191~0.366, FDR_P = 1.17 × 10^−7^; female: rg = 0.085, 95%CI = 0.008~0.167, FDR_P = 1.000). For the female-specific set, we pinpointed three diseases (e.g., K80, male: rg = 0.382, 95%CI = 0.156~0.608, FDR_P = 0.226; female: rg = 0.367, 95%CI = 0.237~0.497, FDR_P = 7.95 × 10^−6^) and seven measurement traits (e.g., trunk fat-free mass, male: rg = 0.150, 95%CI = 0.068~0.232, FDR_P = 0.076; female: rg = 0.250, 95%CI = 0.180~0.320, FDR_P = 7.22 × 10^−10^). Moreover, among 41 overlapped significant results, we also detected 12 sex-specific measurement traits without overlapped 95%CI (e.g., body fat percentage, male: rg = 0.740, 95%CI = 0.675~0.804, FDR_P = 1.81 × 10^−108^; female: rg = 0.457, 95%CI = 0.374~0.540, FDR_P = 1.34 × 10^−24^) (Table 1).

Specifically, we performed local genetic correlation analysis to uncover the distinct loci associated with the two overlapped and three sex-specific correlated diseases across two sexes. We defined 4 significant bivariate loci and 15 nominal bivariate loci for females, as well as 4 significant loci and 4 nominal loci for males (Table S6). For the two overlapped diseases, we found locus 6.52 (chr6: 42103739–43770626, rg = 0.664, 95%CI: 0.453–0.886) and locus 8.37 (chr8: 22895019–23788494, rg = 0.560, 95%CI: 0.256–0.908) showed significant local genetic correlation with chronic ischemic heart disease, but only in the female-specific set. For the three sex-specific correlated diseases, we found locus 14.69 (chr14: 95946793–97174314, rg = 0.529, 95%CI: 0.163–0.957) showed significant local genetic correlation in the female-specific set; locus 17.54 (chr17: 69245591–70495119, rg = −0.755, 95%CI: −1.000~−0.417) and locus 8.93 (chr8: 92876621–94999066, rg = 0.945, 95%CI: 0.367–1.000) showed significant local genetic correlation in the male-specific set with mononeuropathies of upper limb; locus 8.93 (chr8: 92876621–94999066, rg = 0.442, 95%CI: 0.104~0.826) showed significant local genetic correlation only in the male-specific set with cholelithiasis; locus 14.5 (chr14: 23985937–24906056, rg = 0.565, 95%CI: 0.188–1.000) showed significant local genetic correlation in the female-specific set; and locus 20.10 (chr20: 7959829–9279550, rg = −0.475, 95%CI: −0.860~−0.140) showed significant local genetic correlation in the male-specific set with other disorders of the urinary system. Furthermore, we detected no overlapped results across the two sexes for the nominal bivariate loci.

Overall, the results of global and local genetic correlations among two sexes indicate a considerable degree of difference between WHR and complex traits.

### 3.3. Baseline Population Characteristics in UKB

We included a total of 308,373 participants of European ancestry from the UKB. We randomly selected 50,000 people as the validation set to build the PRS model. The remaining 258,373 individuals (138,693 females and 119,680 males) comprised the test set for PRS calculations and subsequent analyses. The baseline characteristics of the 258,373 participants in the test set are presented in Table 2. We observed that the WHR and other covariables were statistically significant across the two sexes.

### 3.4. Sexual Dimorphism for Association of WHR with Outcomes

We observed that WHR had significant associations with 111 traits including 59 diseases and 52 measurement traits in the male-specific set (Table S7), and with 110 traits including 59 diseases and 51 measurement traits in the female-specific set (Table S7). We detected 47.60% (70/147) sex-specific traits, involving 13 traits including 7 diseases (e.g., umbilical hernia, male: OR = 1269.544, 95%CI = 684.268~2355.427, *p* < 1.00 × 10^−400^, female: OR = 3.777, 95%CI = 1.666~8.562, *p* = 0.002) and 6 measurement traits (e.g., leg fat mass (right), male: β = 1.431, 95%CI = 1.363~1.500, *p* < 1.00 × 10^−400^, female: β = 0.053, 95%CI = 0.015~0.090, *p* = 0.006), that were significant only in the male-specific set, and 13 traits including 8 diseases (e.g., mononeuropathies of upper limb, male: OR = 1.829, 95%CI = 1.048–3.194, *p* = 0.034, female: OR = 3.037, 95%CI = 2.151~4.289, *p* = 2.79 × 10^−10^) and 5 measurement traits (e.g., arm fat mass (left), male: β = −0.017, 95%CI = −0.043~0.009, *p* = 0.192, female: β = −0.305, 95%CI = −0.323~−0.287, *p* = 6.29 × 10^−242^) that were significant only in the female-specific set, as well as 44 traits without overlapped 95%CI including 10 diseases (e.g., non-insulin-dependent diabetes mellitus, male: OR = 417.406, 95%CI = 327.164~679.243, *p* = 2.82 × 10^−239^, female: OR = 7941.006, 95%CI = 5584.729~11291.429, *p* < 1.00 × 10^−400^) and 34 measurement traits (e.g., triglycerides, male: β = 3.076, 95%CI = 2.952~3.200, *p* < 1.00 × 10^−400^, female: β = 3.538, 95%CI = 3.471~3.605, *p* < 1.00 × 10^−400^) (Table 3).

### 3.5. Sexual Dimorphism for Association of WHR PGS with Outcomes

**Figure 4:**
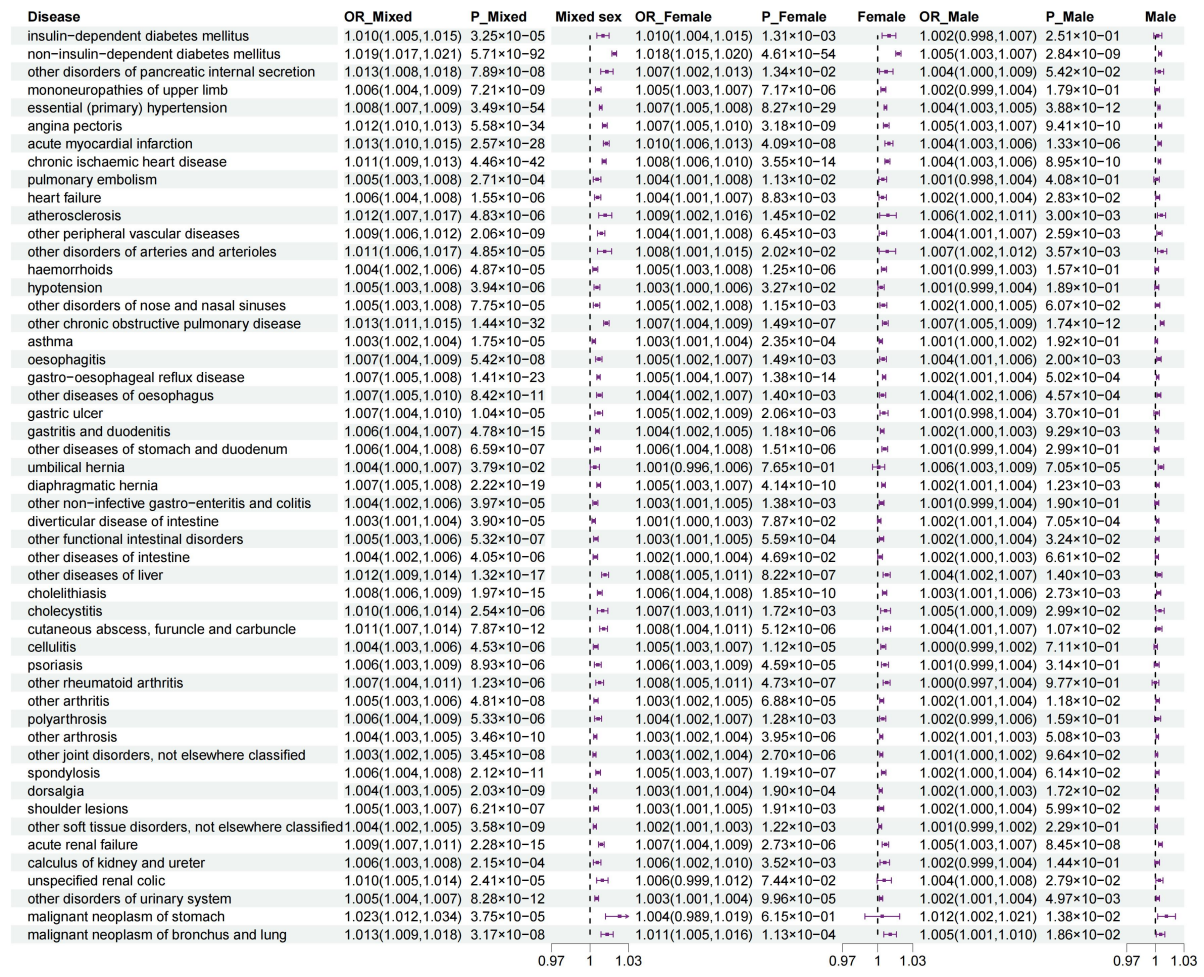
**The relationship of WHR PGS with diseases.** We used the odds ratio (95% CI) to estimate the strength of the association. The forest plots show the β values and *p* values under the different traits for each of the three genders. The WHR PGS had significant associations with 50 diseases for the mixed-sex set, with 27 diseases for the female set, and with 8 diseases for the male set. A total of 23 diseases showed significant sexual dimorphism in association with the WHR PGS, spanning metabolic, cardiovascular, respiratory, musculoskeletal, ocular, and gastrointestinal disorders. The significant threshold was set at < 3.40 × 10^−4^ (0.05/147). CI, confidence interval; WHR, waist-to-hip ratio; PGS, polygenic risk score.

**Figure 5:**
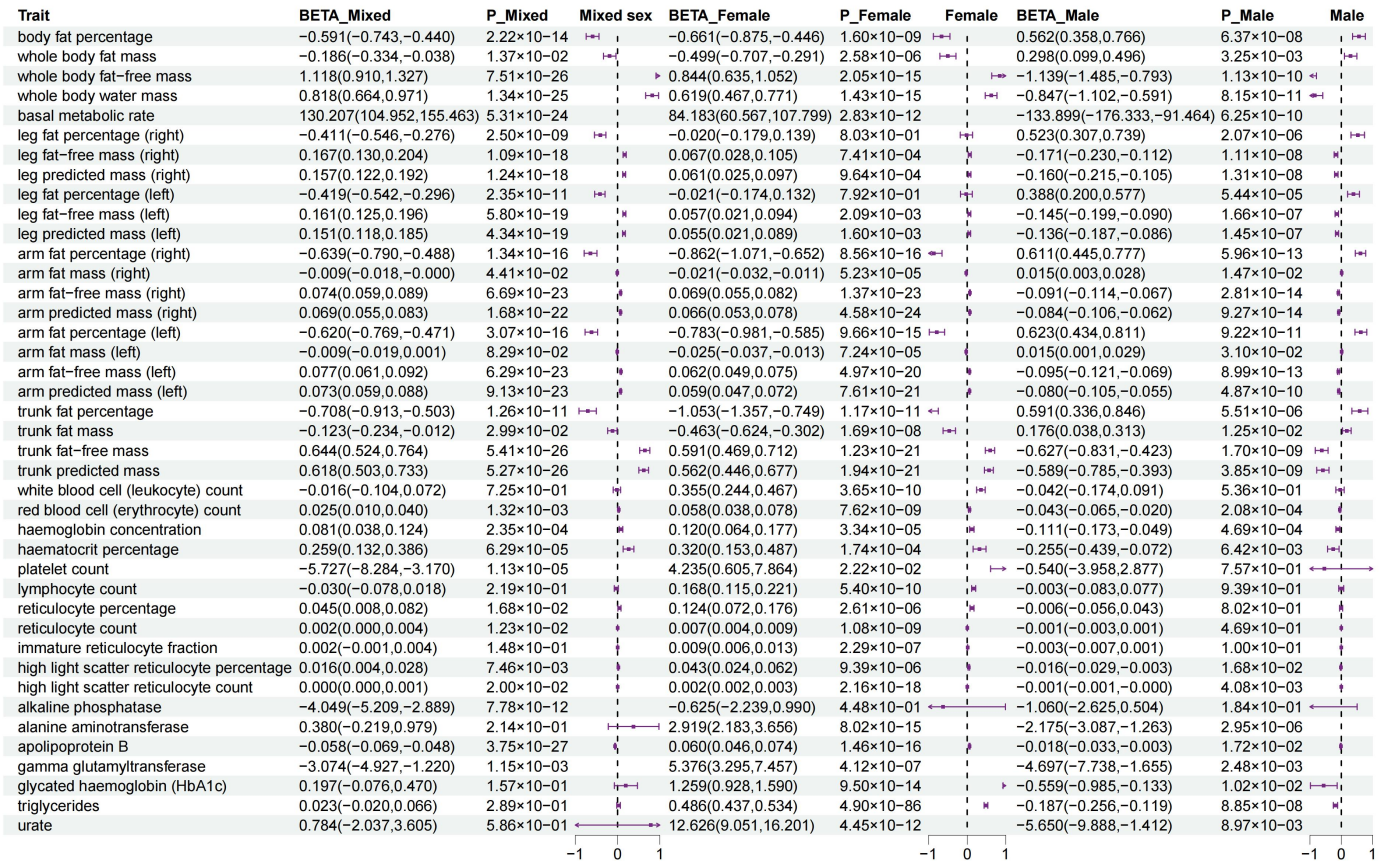
**The relationship of WHR PGS with measurement traits.** We used β (95% CI) to estimate the strength of the association. The forest plots respectively show the β values and *p* values under the different traits for each of the three genders. The WHR PGS had significant associations with 24 measurement traits for the mixed-sex set, with 33 measurement traits for the female set, and with 22 measurement traits for the male set. A total of 22 measurement traits showed significant sexual dimorphism in association with the WHR PGS, involving 19 body measurements, two blood cell counts, and one biochemical marker. Thehe significant threshold was set at < 3.40 × 10^−4^ (0.05/147). CI, confidence interval; WHR, waist-to-hip ratio; PGS, polygenic risk score.

We observed that the WHR PGS had significant associations with 50 diseases for the mixed-sex set, with 27 diseases for the female set, and with 8 diseases for the male set (Figure 4 and Table S8). For measurement traits, we observed significant associations of the WHR PGS with 24 traits in the mixed-sex set, with 33 traits in the female set, and with 22 traits in the male set (Figure 5 and Table S8). Moreover, in the 74 traits significant associations with the WHR PGS in the mixed-sex set, we detected 45 sex-specific traits (60.81%) including 23 diseases and 22 measurement traits (Figure 4 and Figure 5 and Table S9). These diseases involving various disease systems, such as the endocrine system (e.g., non-insulin-dependent diabetes mellitus, female: OR = 1.018, 95%CI = 1.015~1.020, male: OR = 1.005, 95%CI = 1.003~1.007), circulatory system (e.g., essential (primary) hypertension, female: OR = 1.007, 95%CI = 1.005~1.008, male: OR = 1.004, 95%CI = 1.003~1.005); chronic ischemic heart disease (female: OR = 1.008, 95%CI = 1.006~1.010, male: OR = 1.004, 95%CI = 1.003~1.006). and digestive system (e.g., gastroesophageal reflux disease, female: OR = 1.005, 95%CI = 1.004~1.007, male: OR = 1.002, 95%CI = 1.001~1.004).

### 3.6. Sexual Dimorphism for Prediction Models

**Figure 6:**
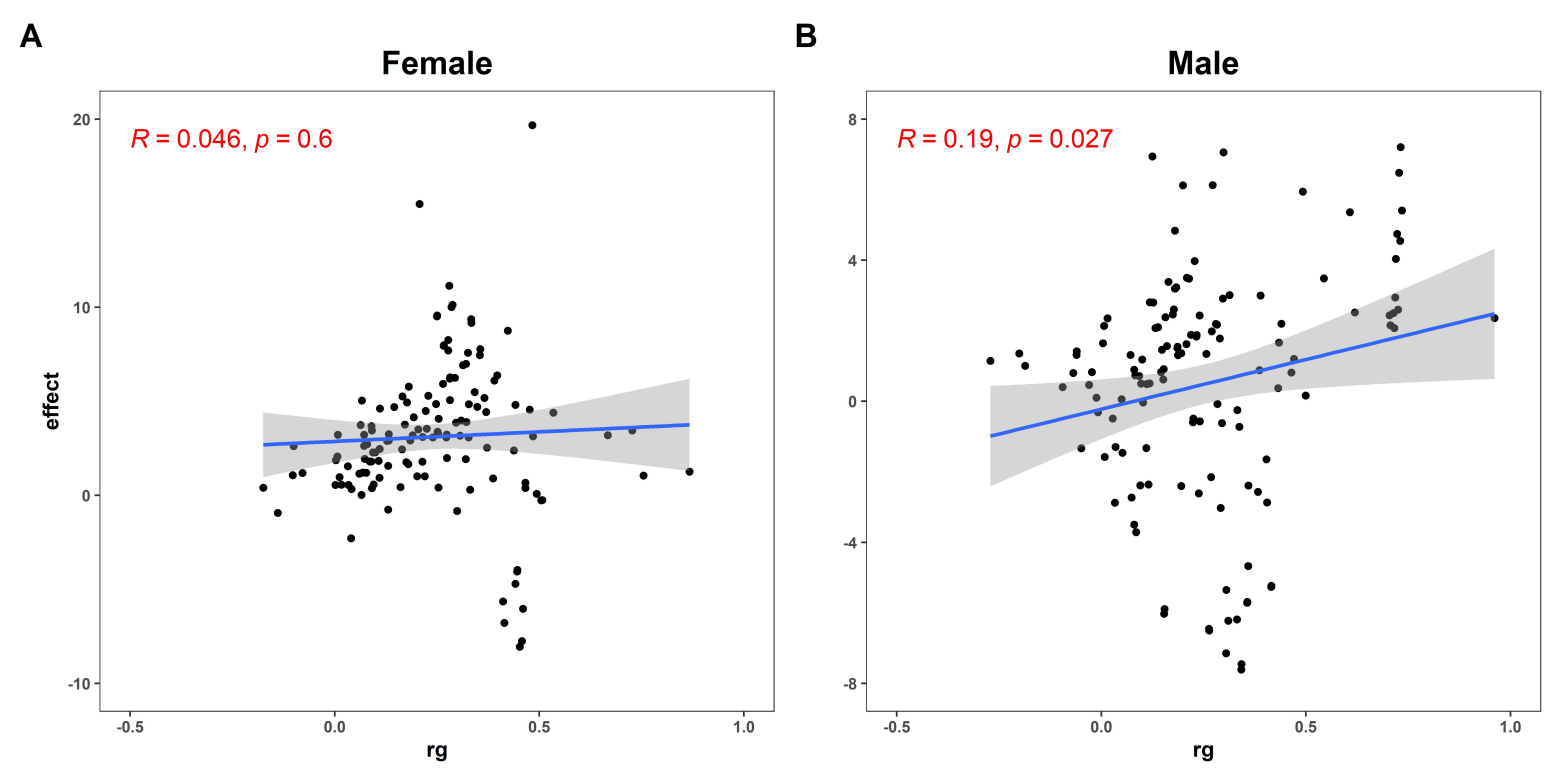
**The relationship between global genetic correlation and the effect value of the regression model.** (**A**) The relationship between global genetic correlation and the effect value of the regression model in the female set; (**B**) The relationship between global genetic correlation and the effect value of the regression model in the male set. The global genetic correlation showed a positive correlation with the effect value for both females and males, and the correlation among males was stronger than that among females. The *x*-axis represents the global genetic association and the *y*-axis represents the effect value of the regression model.

**Figure 7:**
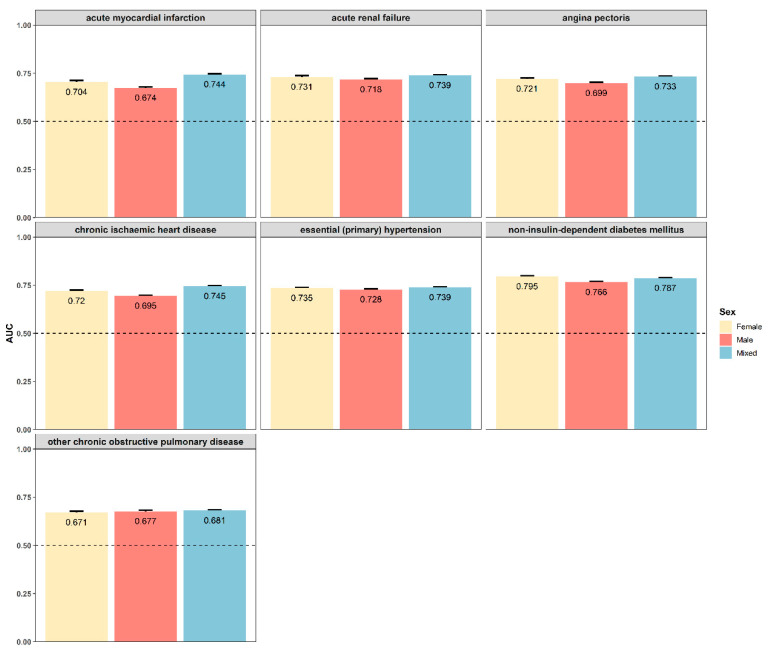
**The prediction of models in different diseases.** We used the facet plots to present the AUC comparison values of the three genders for acute myocardial infarction, acute renal failure, angina pectoris, chronic ischemic heart disease, essential (primary) hypertension, non-insulin-dependent diabetes mellitus, and other chronic obstructive pulmonary diseases. The AUC values ranged from 0.681 to 0.787 in the mixed-sex set, from 0.671 to 0.795 in the female set, and from 0.674 to 0.766 in the male set. The PGS of WHR showed the highest predictive performance for non-insulin-dependent diabetes mellitus in all three genders (0.795 in females, 0.766 in males, and 0.787 in mixed sexes). The *y*-axis represents the AUC value of models. AUC, area under the curve; WHR, waist-to-hip ratio; PGS, polygenic risk score.

We first tested the relationship between global genetic correlation and the effect value of the regression model. We found that the global genetic correlation showed a positive correlation with the effect value for both females (R = 0.046, *p* = 0.600) (Figure 6A) and males (R = 0.190, *p* = 0.027) (Figure 6B). Then we examined traits that were significant and consistently effective with both the WHR and WHR PGS across the three sets. We found seven identical outcomes, all of which belonged to diseases. To test the ability of the WHR PGS to predict these traits, we calculated the AUC value for these traits. We found the AUC values ranged from 0.681 to 0.787 in the mixed-sex set, from 0.671 to 0.795 in the female set, and from 0.674 to 0.766 in the male set (Figure 7). Overall, the WHR PGS had a good predictive effect on these diseases. And the model fitting effect of the female set was better than that of the male set.

## 4. Discussion

Our study explores the genetic structure of WHR differences between sexes, the genetic associations of WHR with complex traits, and the implications for disease risk. We found that the sex specificity of WHR extends beyond its genetic structure to its associations with various complex traits and clinical risks, providing new insights into the sexual dimorphism of WHR. These findings may help refine health management strategies and personalized medicine.

Our analysis revealed notable genetic differences between the sexes in WHR (h^2^ = 0.135 in females, h^2^ = 0.111 in males), confirming and extending prior reports that female body fat distribution is more strongly genetically determined [16]. Through TWAS, we identified *CCDC92* as a female-specific WHR-related gene involved in adipose tissue lipid metabolism, providing a mechanistic explanation for the clinical observation of greater gluteofemoral fat retention in women [38]. In contrast, the male-specific association with *UQCC1*, a gene encoding a mitochondrial respiratory chain component, suggests that fundamental differences in energy utilization may underlie sex-specific fat distribution patterns [39]. These findings not only demonstrate sex-differential effects at previously identified loci [40,41] but also provide critical functional context through tissue-specific expression analyses, revealing pathway-level differences between sexes and significantly expanding upon prior genome-wide association studies.

Our genetic correlation, cross-sectional, and PGS analyses revealed sex-specific associations between WHR and multi-system chronic diseases as well as measurements. Specifically, sex-stratified analyses identified diseases spanning metabolic, cardiovascular, respiratory, musculoskeletal, ocular, and gastrointestinal disorders. Additionally, WHR exhibited sex-differentiated correlations with body fat distribution, blood cell parameters, and blood biochemical markers.

For metabolic diseases, we observed an obvious association between WHR and type 2 diabetes (T2D), and this association was stronger in women, which is consistent with the results of many previous studies [17,42,43,44]. This likely reflects sex differences in adipose tissue expandability [45], ectopic fat deposition patterns [46], and hormonal regulation of insulin sensitivity [47]. In the spectrum of cardiovascular diseases, the association between WHR and acute/chronic ischemic heart disease showed similar sex-specific patterns. Our findings aligned with the conclusions of studies by Yusuf et al. [48] and Emdin et al. [44], but we provided more direct causal evidence through genetic analysis. Notably, we first confirmed a significant sex difference in the genetic association between WHR and peripheral vascular disease (male: β = 2.929, female: statistically non-significant), suggesting that this may explain the clinical observation that men are more prone to peripheral artery disease [49]. Moreover, local genetic correlation analysis identified several loci including locus 6.52 (chr6: 42103739–43770626) and locus 8.37 (chr8: 22895019–23788494) showing female-specific associations between WHR and chronic ischemic heart disease. Key genes within locus 6.52, including *VEGFA*, *SRF*, and *GNMT*, established a link between obesity and chronic ischemic heart disease. These genes may jointly influence the mechanisms of these two diseases through anti-angiogenesis [50,51], nuclear transcription [52], and DNA methylation [53]. The analysis of respiratory diseases identified unexpected findings: the association between WHR and chronic obstructive pulmonary disease (COPD) and asthma was significantly stronger in males. This is consistent with clinical observations reported by Breyer et al. [54] and Chen et al. [55], but opposite to the findings of Camp et al. [56] and Santos et al. [57]. Our findings suggest potential sex-specific differences in how fat distribution influences respiratory function. Notably, our genetic evidence supports that males may be more susceptible to obesity-related asthma due to abdominal fat accumulation [58]. The musculoskeletal disease analysis obtained novel findings. WHR showed a positive association with coxarthrosis in females but a negative association in males. Previous studies have found the association in females is stronger than that in males [59], but our sex-stratified analysis provided the first clear evidence of this directional divergence. Similarly, knee osteoarthritis analysis revealed significant sex-specific effects, offering genetic insights into clinically observed sex differences in obesity-related osteoarthritis [60]. The analysis of ocular diseases revealed a stronger association between WHR and age-related cataracts in males compared to females, which is consistent with findings reported by Lou et al. [61]. The mechanism may be related to the protective effect of estrogen on the lens [62]. As for the association between WHR and the gastrointestinal tract, our findings showed generally stronger effects in females for conditions like gastro-esophageal reflux disease and ulcers [63,64], while hernias showed male-predominant association [65]. These patterns may relate to sex differences in visceral adipose inflammation or mechanical effects on abdominal organs [66].

For measurement indicators, in terms of body measurements, our study confirmed strong associations between WHR and both total and regional fat mass. Notably, men exhibited significantly higher effect sizes than women for trunk fat percentage and total mass. These findings were highly consistent with the multicenter study by Fox et al. [67], but our results provided more precise quantification. The sex-specific patterns in limb fat distribution appeared more complex, potentially reflecting hormonal regulation of regional fat deposition [45]. Our analysis on blood indicators revealed systemic associations between WHR and multiple blood cell parameters. The sex-specific patterns in white blood cell (WBC) and neutrophil counts corresponded to the findings from Furman et al. [68], supporting the link between abdominal obesity and low-grade chronic inflammation. Notably, the stronger association between WHR and monocyte counts in males may reflect sex differences in adipose tissue macrophage infiltration [69]. In terms of blood biochemical markers, our study not only confirmed known associations between WHR and liver enzymes/lipid profiles [70,71], but also revealed significant sex differences. Specifically, γ glutamyltransferase showed stronger effects in males than females, aligning with sex-specific patterns in non-alcoholic fatty liver disease epidemiology [70]. Conversely, WHR demonstrated a stronger association with uric acid levels in females, potentially explaining the increased gout risk observed in postmenopausal women [72].

PGSs are crucial for deciphering complex genetic traits and advancing precision medicine [73,74,75]. Our PGS prediction models for WHR demonstrated high accuracy in disease risk prediction. Among them, the prediction of T2D reached nearly 0.8, with better performance in females than males. These key findings not only provide new molecular evidence for sex dimorphism in WHR-related genetic effects but also establish a theoretical basis for sex-specific risk assessment and precision prevention strategies for obesity-related diseases.

In summary, our systematic genetic analyses not only verify previous epidemiological findings but, more importantly, uncover distinct sex-differential patterns underlying these associations. These discoveries will provide novel insights into the pathogenesis of obesity-related diseases, particularly highlighting the critical need to consider sex differences in clinical assessment and intervention strategies. Compared to prior studies, our genetic approach offers stronger evidence for causal inference, and large-scale sex-stratified analyses reveal many previously underappreciated sex-specific associations. These findings may provide personalized obesity management strategies for precision medicine.

## 5. Limitation

First, our study was limited to individuals of European ancestry, necessitating further validation in populations of diverse ethnic backgrounds to enhance the generalizability of the findings and promote health equity. Second, for the UKB individual-level data, we only relied on baseline survey data to define each participant’s WHR and covariates, which may have resulted in failure to accurately capture potential associations with changes in these measures over time. Third, the traits included in our analysis were limited, potentially overlooking associations between WHR and certain unexamined traits. Fourth, the lack of external data for validation in our modeling analyses may affect the robustness of the results. Finally, the cross-sectional analysis limits its capacity to infer causality. Although we examined the associations of WHR and WHR PRS with complex traits, more complete research designs are still required to provide stronger causal evidence.

## 6. Conclusions

Our study identified the sexual dimorphism of WHR and the differences in the association and risk of WHR with complex traits between sexes. Our findings not only advance the biological understanding of WHR-related sex differences but also provide valuable insights for health management, sociocultural analysis, and clinical medicine.

## Figures and Tables

**Table 1:** The global genetic correlation of WHR with sex-specific traits.

Description	Female		Male	
	rg (95%CI)	FDR_P	rg (95%CI)	FDR_P
mononeuropathies of upper limb	0.234 (0.139, 0.328)	3.63 × 10^−4^	0.269 (0.083, 0.455)	1
cholelithiasis	0.367 (0.237, 0.497)	7.95 × 10^−6^	0.382 (0.157, 0.608)	0.227
other disorders of urinary system	0.316 (0.199, 0.433)	2.97 × 10^−5^	0.039 (−0.262, 0.34)	1
body fat percentage	0.457 (0.374, 0.54)	1.34 × 10^−24^	0.74 (0.675, 0.804)	1.81 × 10^−108^
whole body fat mass	0.439 (0.358, 0.52)	9.05 × 10^−24^	0.723 (0.654, 0.792)	1.85 × 10^−91^
leg fat percentage (right)	0.504 (0.43, 0.577)	5.35 × 10^−39^	0.728 (0.663, 0.794)	2.01 × 10^−102^
leg fat mass (right)	0.463 (0.385, 0.541)	5.01 × 10^−29^	0.732 (0.663, 0.8)	1.27 × 10^−94^
leg fat percentage (left)	0.5 (0.427, 0.574)	5.63 × 10^−38^	0.726 (0.661, 0.791)	1.02 × 10^−103^
leg fat mass (left)	0.463 (0.385, 0.541)	9.48 × 10^−29^	0.723 (0.654, 0.792)	4.53 × 10^−92^
arm fat percentage (right)	0.448 (0.367, 0.529)	7.28 × 10^−25^	0.735 (0.666, 0.803)	8.20 × 10^−96^
arm fat mass (right)	0.444 (0.364, 0.523)	4.21 × 10^−25^	0.709 (0.637, 0.781)	4.17 × 10^−80^
arm fat percentage (left)	0.453 (0.372, 0.534)	1.15 × 10^−25^	0.73 (0.661, 0.798)	5.87 × 10^−94^
arm fat mass (left)	0.443 (0.363, 0.524)	1.53 × 10^−24^	0.71 (0.637, 0.783)	3.65 × 10^−78^
trunk fat percentage	0.412 (0.325, 0.498)	3.34 × 10^−18^	0.735 (0.67, 0.8)	3.45 × 10^−105^
trunk fat mass	0.409 (0.326, 0.493)	2.43 × 10^−19^	0.717 (0.648, 0.785)	9.80 × 10^−91^
trunk fat-free mass	0.25 (0.18, 0.32)	7.22 × 10^−10^	0.15 (0.068, 0.232)	0.076
trunk predicted mass	0.25 (0.18, 0.32)	5.74 × 10^−10^	0.15 (0.068, 0.232)	0.076
red blood cell (erythrocyte) count	0.18 (0.123, 0.236)	1.23 × 10^−7^	0.086 (0.001, 0.171)	1
haemoglobin concentration	0.202 (0.131, 0.274)	6.88 × 10^−6^	0.07 (−0.014, 0.155)	1
haematocrit percentage	0.174 (0.105, 0.242)	1.49 × 10^−4^	0.069 (−0.019, 0.156)	1
monocyte count	0.085 (0.004, 0.167)	1	0.278 (0.191, 0.366)	1.17 × 10^−7^
apolipoprotein B	0.278 (0.141, 0.414)	0.017	0.098 (−0.036, 0.232)	1

Notes: The sex-specific traits including three diseases and 19 measurement traits showing global genetic correlation with WHR. Three diseases and 8 measurement traits were only significant in either the female-specific or male-specific set, while 12 sex-specific measurement traits showed non-overlapping 95%CI. WHR, waist-to-hip ratio; CI, confidence interval.

**Table 2:** Baseline characteristics of participants from the UK Biobank.

Variable	All (N = 258,637)	Female (N = 139,063)	Male (N = 119,574)	*p*
age	56.64 ± 8.01	56.44 ± 7.91	56.88 ± 8.13	<0.001
WHR	0.87 ± 0.09	0.82 ± 0.07	0.93 ± 0.07	<0.001
BMI	27.34 ± 4.78	26.95 ± 5.16	27.80 ± 4.22	<0.001
sleep	7.17 ± 1.08	7.19 ± 1.09	7.14 ± 1.07	<0.001
TDI	−1.47 ± 3.00	−1.50 ± 2.95	−1.44 ± 3.06	<0.001
smoking				<0.001
never	139,819 (54.1%)	81,397 (58.8%)	58,154 (48.6%)	
past	91,773 (35.5%)	45,050 (32.4%)	46,792 (39.1%)	
current	26,781 (10.4%)	12,246 (8.8%)	14,628 (12.2%)	
drinking				<0.001
never	8074 (3.1%)	5962 (4.3%)	2067 (1.7%)	
past	8943 (3.5%)	4989 (3.6%)	3959 (3.3%)	
current	241,356 (93.4%)	127,742 (92.1%)	113,548 (95.0%)	
activity	109.29 ± 101.81	105.71 ± 92.82	113.45 ± 111.44	<0.001

Notes: These are the baseline characteristics of participants in our study. There were significant differences in age, WHR, BMI, sleep, TSM, smoking, drinking, and activity between females and males (*p* < 0.001). WHR, waist-to-hip ratio; BMI, body mass index; TDI, Townsend Deprivation Index.

**Table 3:** The cross-sectional association of WHR with sex-specific traits.

Description	Female		Male	
	β (95%CI)	*p*	β (95%CI)	*p*
malignant neoplasm of colon	0.246 (−0.611, 1.103)	0.573	1.762 (0.846, 2.679)	1.63 × 10^−4^
non-insulin-dependent diabetes mellitus	8.98 (8.628, 9.332)	<1.00 × 10^−400^	6.156 (5.79, 6.521)	2.82 × 10^−239^
mononeuropathies of upper limb	1.111 (0.766, 1.456)	2.79 × 10^−10^	0.604 (0.047, 1.161)	0.034
senile cataract	0.54 (0.214, 0.865)	0.001	1.405 (0.952, 1.857)	1.18 × 10^−9^
other cataract	0.363 (0.087, 0.638)	0.010	1.011 (0.638, 1.385)	1.13 × 10^−7^
acute myocardial infarction	3.475 (2.934, 4.016)	2.71 × 10^−36^	2.32 (1.919, 2.722)	1.00 × 10^−29^
chronic ischemic heart disease	3.294 (2.95, 3.637)	1.08 × 10^−78^	2.454 (2.148, 2.759)	6.99 × 10^−56^
other peripheral vascular diseases	0.173 (−0.375, 0.722)	0.535	2.929 (2.325, 3.532)	1.86 × 10^−21^
arterial embolism and thrombosis	2.821 (1.277, 4.365)	3.42 × 10^−4^	4.817 (3.662, 5.972)	2.97 × 10^−16^
haemorrhoids	1.15 (0.789, 1.512)	4.52 × 10^−10^	0.453 (0.008, 0.898)	0.046
nasal polyp	1.107 (0.274, 1.939)	0.009	2.343 (1.599, 3.086)	6.53 × 10^−10^
other chronic obstructive pulmonary disease	3.65 (3.244, 4.057)	2.59 × 10^−69^	5.301 (4.844, 5.758)	1.81 × 10^−114^
asthma	2.05 (1.815, 2.285)	1.51 × 10^−65^	3.079 (2.755, 3.402)	1.16 × 10^−77^
umbilical hernia	1.329 (0.51, 2.147)	0.001	7.146 (6.528, 7.764)	1.05 × 10^−113^
ventral hernia	1.584 (0.894, 2.273)	6.72 × 10^−6^	4.095 (3.33, 4.86)	9.04 × 10^−26^
diverticular disease of intestine	2.061 (1.818, 2.304)	3.79 × 10^−62^	2.74 (2.431, 3.049)	1.17 × 10^−67^
other diseases of intestine	1.678 (1.343, 2.014)	9.64 × 10^−23^	2.419 (2.042, 2.795)	2.28 × 10^−36^
polyarthrosis	0.968 (0.546, 1.391)	7.11 × 10^−6^	0.966 (0.266, 1.666)	0.007
coxarthrosis [arthrosis of hip]	0.65 (0.307, 0.993)	2.03 × 10^−4^	−1.449 (−1.941, −0.957)	7.76 × 10^−9^
other arthrosis	0.547 (0.33, 0.764)	8.00 × 10^−7^	0.124 (−0.189, 0.437)	0.437
acquired deformities of fingers and toes	−0.778 (−1.127, −0.429)	1.27 × 10^−5^	−0.454 (−1.292, 0.385)	0.289
internal derangement of knee	−0.021 (−0.458, 0.415)	0.924	−1.531 (−2.008, −1.054)	3.19 × 10^−10^
other joint disorders, not elsewhere classified	0.502 (0.285, 0.719)	5.75 × 10^−6^	0.295 (0.001, 0.588)	0.049
spondylosis	0.675 (0.361, 0.989)	2.52 × 10^−5^	0.601 (0.158, 1.045)	0.008
other disorders of bladder	0.911 (0.436, 1.385)	1.69 × 10^−4^	0.335 (−0.133, 0.804)	0.161
body fat percentage	7.149 (6.838, 7.459)	<1.00 × 10^−400^	17.066 (16.703, 17.43)	<1.00 × 10^−400^
whole body fat mass	2.15 (1.847, 2.452)	5.16 × 10^−44^	11.007 (10.646, 11.367)	<1.00 × 10^−400^
whole body fat-free mass	−2.031 (−2.336, −1.726)	6.72 × 10^−39^	−9.63 (−10.269, −8.992)	1.80 × 10^−191^
whole body water mass	−1.53 (−1.753, −1.308)	2.65 × 10^−41^	−7.281 (−7.751, −6.811)	1.73 × 10^−201^
basal metabolic rate	−200.554 (−235.152, −165.956)	6.70 × 10^−30^	−1035.291 (−1113.559, −957.023)	8.86 × 10^−148^
leg fat percentage (right)	7.339 (7.11, 7.567)	<1.00 × 10^−400^	15.18 (14.791, 15.569)	<1.00 × 10^−400^
leg fat mass (right)	0.053 (0.015, 0.09)	0.006	1.431 (1.363, 1.5)	<1.00 × 10^−400^
leg fat-free mass (right)	−0.886 (−0.943, −0.83)	5.06 × 10^−207^	−1.875 (−1.983, −1.767)	6.44 × 10^−253^
leg predicted mass (right)	−0.831 (−0.884, −0.778)	3.25 × 10^−207^	−1.76 (−1.861, −1.659)	2.90 × 10^−253^
leg fat percentage (left)	7.088 (6.869, 7.308)	<1.00 × 10^−400^	13.018 (12.678, 13.358)	<1.00 × 10^−400^
leg fat mass (left)	0.049 (0.011, 0.086)	0.010	1.211 (1.149, 1.273)	1.11 × 10^−308^
leg fat-free mass (left)	−0.801 (−0.855, −0.748)	1.93 × 10^−189^	−1.357 (−1.457, −1.257)	1.21 × 10^−155^
leg predicted mass (left)	−0.75 (−0.8, −0.7)	3.26 × 10^−189^	−1.271 (−1.365, −1.177)	2.59 × 10^−155^
arm fat percentage (right)	5.735 (5.43, 6.039)	8.09 × 10^−297^	9.029 (8.726, 9.331)	<1.00 × 10^−400^
arm fat mass (right)	−0.209 (−0.224, −0.194)	4.39 × 10^−164^	0.003 (−0.02, 0.025)	0.824
arm fat-free mass (right)	−0.001 (−0.02, 0.019)	0.953	−0.57 (−0.613, −0.527)	2.49 × 10^−148^
arm predicted mass (right)	0.002 (−0.017, 0.02)	0.871	−0.535 (−0.575, −0.494)	9.25 × 10^−147^
arm fat percentage (left)	5.546 (5.258, 5.833)	1.11 × 10^−308^	10.24 (9.898, 10.582)	<1.00 × 10^−400^
arm fat mass (left)	−0.305 (−0.323, −0.287)	6.29 × 10^−242^	−0.017 (−0.043, 0.009)	0.192
arm fat-free mass (left)	0.004 (−0.016, 0.023)	0.721	−0.7 (−0.748, −0.652)	3.59 × 10^−178^
arm predicted mass (left)	0.001 (−0.017, 0.019)	0.934	−0.655 (−0.701, −0.608)	1.61 × 10^−167^
trunk fat percentage	7.784 (7.343, 8.225)	1.64 × 10^−261^	20.262 (19.806, 20.718)	<1.00 × 10^−400^
trunk fat mass	2.564 (2.33, 2.798)	3.63 × 10^−102^	8.515 (8.267, 8.764)	<1.00 × 10^−400^
trunk fat-free mass	−0.35 (−0.527, −0.173)	1.08 × 10^−4^	−5.173 (−5.549, −4.797)	5.02 × 10^−160^
trunk predicted mass	−0.324 (−0.494, −0.155)	1.75 × 10^−4^	−4.928 (−5.289, −4.567)	2.63 × 10^−157^
white blood cell (leukocyte) count	2.707 (2.55, 2.864)	8.99 × 10^−250^	3.624 (3.388, 3.86)	2.12 × 10^−198^
red blood cell (erythrocyte) count	0.384 (0.355, 0.413)	3.32 × 10^−152^	0.493 (0.452, 0.534)	2.61 × 10^−121^
haemoglobin concentration	1.077 (0.996, 1.159)	9.19 × 10^−147^	1.494 (1.381, 1.608)	4.00 × 10^−147^
haematocrit percentage	2.622 (2.381, 2.862)	7.14 × 10^−101^	4.084 (3.749, 4.419)	9.44 × 10^−126^
mean corpuscular haemoglobin concentration	0.425 (0.331, 0.52)	1.22 × 10^−18^	0.182 (0.062, 0.303)	0.003
red blood cell (erythrocyte) distribution width	−0.19 (−0.28, −0.101)	3.02 × 10^−5^	0.133 (0.037, 0.229)	0.007
platelet count	66.517 (61.26, 71.774)	1.82 × 10^−135^	51.692 (45.434, 57.949)	6.85 × 10^−59^
monocyte count	0.148 (0.129, 0.167)	3.21 × 10^−53^	0.273 (0.248, 0.299)	1.46 × 10^−99^
neutrophill count	1.69 (1.572, 1.807)	5.98 × 10^−174^	2.666 (2.51, 2.822)	3.67 × 10^−244^
high light scatter reticulocyte percentage	0.532 (0.505, 0.56)	1.11 × 10^−308^	0.471 (0.447, 0.494)	<1.00 × 10^−400^
alanine aminotransferase	25.952 (24.892, 27.013)	<1.00 × 10^−400^	29.995 (28.333, 31.657)	1.36 × 10^−272^
apolipoprotein B	0.429 (0.408, 0.449)	<1.00 × 10^−400^	0.22 (0.193, 0.247)	1.41 × 10^−57^
aspartate aminotransferase	9.516 (8.651, 10.381)	7.50 × 10^−103^	6.946 (5.655, 8.236)	5.21 × 10^−26^
urea	0.209 (0.097, 0.32)	2.44 × 10^−4^	−0.066 (−0.227, 0.095)	0.419
calcium	0.088 (0.079, 0.097)	2.83 × 10^−83^	0.058 (0.047, 0.068)	1.11 × 10^−26^
cystatin C	0.08 (0.068, 0.093)	1.51 × 10^−35^	0.202 (0.183, 0.22)	5.27 × 10^−97^
γ glutamyltransferase	57.233 (54.232, 60.235)	4.59 × 10^−304^	88.281 (82.749, 93.812)	6.85 × 10^−214^
glucose	1.496 (1.401, 1.591)	2.13 × 10^−209^	1.794 (1.636, 1.952)	2.51 × 10^−109^
triglycerides	3.538 (3.471, 3.605)	<1.00 × 10^−400^	3.076 (2.952, 3.2)	<1.00 × 10^−400^
urate	133.014 (127.882, 138.147)	<1.00 × 10^−400^	104.943 (97.223, 112.663)	6.40 × 10^−156^

Notes: The logistic analysis identified 70 sex-specific traits including 25 diseases and 45 measurement traits showing association with WHR. A total of 13 traits including 7 diseases and 6 measurement traits showed significance only in the male-specific set, and 13 traits including 8 diseases and 5 measurement traits showed significance only in the female-specific set, while there were 44 traits without overlapping 95%CI including 10 diseases and 34 measurement traits. *p* < 3.40 × 10^−4^ (0.05/147) was considered significant. WHR, waist-to-hip ratio; CI, confidence interval.

## Data Availability

The GWAS summary statistics for WHR were from the GIANT consortium (https://portals.broadinstitute.org/collaboration/giant/ (accessed on 25 September 2024)). The GWAS summary statistics for complex traits were from the Neale Lab (http://www.nealelab.is/uk-biobank (accessed on 25 September 2024)). The individual data was from the UKB (https://www.ukbiobank.ac.uk/ (accessed on 14 September 2024)). The predefined loci file was downloaded from https://github.com/josefin-werme/LAVA/tree/main/support_data (accessed on 16 December 2024). The tissue-specific TWAS weights on GTEx V8 EUR individuals were downloaded from http://gusevlab.org/projects/fusion/#gtex-v8-multi-tissue-expression (accessed on 11 November 2024).

## Supplementary Materials

The following supporting information can be downloaded at: https://www.mdpi.com/article/10.3390/genes16060711/s1, Table S1. Information about GWAS summary statistics of UKB phenotypes. Table S2. The sex-specific genes of TWAS. Table S3. Traits with nominal genetic correlations with WHR in the mixed-sex set (*p* < 0.05). Table S4. Traits with significant genetic correlations with WHR in the male set (Bonferroni-adjusted *p* < 0.05). Table S5. Traits with significant genetic correlations with WHR in the female set (Bonferroni-adjusted *p* < 0.05). Table S6. The sex-specific local shared loci of five diseases with WHR. Table S7. The relationship of WHR with complex traits in females and males. Table S8. The relationship of WHR PGS with complex traits in females and males. Table S9. The relationship of WHR PGS with sex-specific traits. Figure S1. TWAS results of WHR in adipose visceral omentum tissue. Figure S2. TWAS results of WHR in whole blood tissue.

## References

[1] Bray G.A., Kim K.K., Wilding J.P.H. (2017). Obesity: A chronic relapsing progressive disease process. A position statement of the World Obesity Federation. Obes. Rev..

[2] Roberto C.A., Swinburn B., Hawkes C., Huang T.T., Costa S.A., Ashe M., Zwicker L., Cawley J.H., Brownell K.D. (2015). Patchy progress on obesity prevention: Emerging examples, entrenched barriers, and new thinking. Lancet.

[3] Afshin A., Forouzanfar M.H., Reitsma M.B., Sur P., Estep K., Lee A., Marczak L., Mokdad A.H., Moradi-Lakeh M., Naghavi M. (2017). Health Effects of Overweight and Obesity in 195 Countries over 25 Years. N. Engl. J. Med..

[4] Finucane M.M., Stevens G.A., Cowan M.J., Danaei G., Lin J.K., Paciorek C.J., Singh G.M., Gutierrez H.R., Lu Y., Bahalim A.N. (2011). National, regional, and global trends in body-mass index since 1980: Systematic analysis of health examination surveys and epidemiological studies with 960 country-years and 9·1 million participants. Lancet.

[5] Husain M.J., Datta B.K., Kostova D., Joseph K.T., Asma S., Richter P., Jaffe M.G., Kishore S.P. (2020). Access to Cardiovascular Disease and Hypertension Medicines in Developing Countries: An Analysis of Essential Medicine Lists, Price, Availability, and Affordability. J. Am. Heart Assoc..

[6] Lobstein T., Baur L., Uauy R. (2004). Obesity in children and young people: A crisis in public health. Obes. Rev..

[7] Dashti H.S., Miranda N., Cade B.E., Huang T., Redline S., Karlson E.W., Saxena R. (2022). Interaction of obesity polygenic score with lifestyle risk factors in an electronic health record biobank. BMC Med..

[8] Flegal K.M., Kit B.K., Orpana H., Graubard B.I. (2013). Association of all-cause mortality with overweight and obesity using standard body mass index categories: A systematic review and meta-analysis. Jama.

[9] Zimmet P.Z., Alberti K.G. (2006). Introduction: Globalization and the non-communicable disease epidemic. Obesity.

[10] Salmón-Gómez L., Catalán V., Frühbeck G., Gómez-Ambrosi J. (2023). Relevance of body composition in phenotyping the obesities. Rev. Endocr. Metab. Disord..

[11] Ebrahimzadeh Attari V., Nourmohammadi M., Asghari-Jafarabadi M., Mahluji S., Malek Mahdavi A., Esmaeili P. (2024). Prediction the changes of anthropometric indices following a weight-loss diet in overweight and obese women by mathematical models. Sci. Rep..

[12] Wells J.C. (2007). Sexual dimorphism of body composition. Best. Pract. Res. Clin. Endocrinol. Metab..

[13] Pawłowski B., Dunbar R.I. (2005). Waist-to-hip ratio versus body mass index as predictors of fitness in women. Hum. Nat..

[14] Karastergiou K., Smith S.R., Greenberg A.S., Fried S.K. (2012). Sex differences in human adipose tissues—The biology of pear shape. Biol. Sex. Differ..

[15] Heid I.M., Jackson A.U., Randall J.C., Winkler T.W., Qi L., Steinthorsdottir V., Thorleifsson G., Zillikens M.C., Speliotes E.K., Mägi R. (2010). Meta-analysis identifies 13 new loci associated with waist-hip ratio and reveals sexual dimorphism in the genetic basis of fat distribution. Nat. Genet..

[16] Pulit S.L., Stoneman C., Morris A.P., Wood A.R., Glastonbury C.A., Tyrrell J., Yengo L., Ferreira T., Marouli E., Ji Y. (2019). Meta-analysis of genome-wide association studies for body fat distribution in 694 649 individuals of European ancestry. Hum. Mol. Genet..

[17] Randall J.C., Winkler T.W., Kutalik Z., Berndt S.I., Jackson A.U., Monda K.L., Kilpeläinen T.O., Esko T., Mägi R., Li S. (2013). Sex-stratified genome-wide association studies including 270,000 individuals show sexual dimorphism in genetic loci for anthropometric traits. PLoS Genet..

[18] Shungin D., Winkler T.W., Croteau-Chonka D.C., Ferreira T., Locke A.E., Mägi R., Strawbridge R.J., Pers T.H., Fischer K., Justice A.E. (2015). New genetic loci link adipose and insulin biology to body fat distribution. Nature.

[19] Pedersen S.B., Kristensen K., Hermann P.A., Katzenellenbogen J.A., Richelsen B. (2004). Estrogen controls lipolysis by up-regulating alpha2A-adrenergic receptors directly in human adipose tissue through the estrogen receptor α. Implications for the female fat distribution. J. Clin. Endocrinol. Metab..

[20] Censin J.C., Peters S.A.E., Bovijn J., Ferreira T., Pulit S.L., Mägi R., Mahajan A., Holmes M.V., Lindgren C.M. (2019). Causal relationships between obesity and the leading causes of death in women and men. PLoS Genet..

[21] Marees A.T., de Kluiver H., Stringer S., Vorspan F., Curis E., Marie-Claire C., Derks E.M. (2018). A tutorial on conducting genome-wide association studies: Quality control and statistical analysis. Int. J. Methods Psychiatr. Res..

[22] Sudlow C., Gallacher J., Allen N., Beral V., Burton P., Danesh J., Downey P., Elliott P., Green J., Landray M. (2015). UK biobank: An open access resource for identifying the causes of a wide range of complex diseases of middle and old age. PLoS Med..

[23] Katzmarzyk P.T., Lee I.M., Martin C.K., Blair S.N. (2017). Epidemiology of Physical Activity and Exercise Training in the United States. Prog. Cardiovasc. Dis..

[24] Yang S., Zhou X. (2020). Accurate and Scalable Construction of Polygenic Scores in Large Biobank Data Sets. Am. J. Hum. Genet..

[25] Yang S., Zhou X. (2022). PGS-server: Accuracy, robustness and transferability of polygenic score methods for biobank scale studies. Brief. Bioinform..

[26] Ye X., Wang Y., Zou Y., Tu J., Tang W., Yu R., Yang S., Huang P. (2023). Associations of socioeconomic status with infectious diseases mediated by lifestyle, environmental pollution and chronic comorbidities: A comprehensive evaluation based on UK Biobank. Infect. Dis. Poverty.

[27] Gusev A., Ko A., Shi H., Bhatia G., Chung W., Penninx B.W., Jansen R., de Geus E.J., Boomsma D.I., Wright F.A. (2016). Integrative approaches for large-scale transcriptome-wide association studies. Nat. Genet..

[28] Wainberg M., Sinnott-Armstrong N., Mancuso N., Barbeira A.N., Knowles D.A., Golan D., Ermel R., Ruusalepp A., Quertermous T., Hao K. (2019). Opportunities and challenges for transcriptome-wide association studies. Nat. Genet..

[29] Beaglehole R., Ebrahim S., Reddy S., Voûte J., Leeder S. (2007). Prevention of chronic diseases: A call to action. Lancet.

[30] Bulik-Sullivan B.K., Loh P.R., Finucane H.K., Ripke S., Yang J., Patterson N., Daly M.J., Price A.L., Neale B.M. (2015). LD Score regression distinguishes confounding from polygenicity in genome-wide association studies. Nat. Genet..

[31] Auton A., Brooks L.D., Durbin R.M., Garrison E.P., Kang H.M., Korbel J.O., Marchini J.L., McCarthy S., McVean G.A., Abecasis G.R. (2015). A global reference for human genetic variation. Nature.

[32] Werme J., van der Sluis S., Posthuma D., de Leeuw C.A. (2022). An integrated framework for local genetic correlation analysis. Nat. Genet..

[33] Bulik-Sullivan B., Finucane H.K., Anttila V., Gusev A., Day F.R., Loh P.R., Duncan L., Perry J.R., Patterson N., Robinson E.B. (2015). An atlas of genetic correlations across human diseases and traits. Nat. Genet..

[34] Gerring Z.F., Thorp J.G., Gamazon E.R., Derks E.M. (2022). A Local Genetic Correlation Analysis Provides Biological Insights Into the Shared Genetic Architecture of Psychiatric and Substance Use Phenotypes. Biol. Psychiatry.

[35] Privé F., Vilhjálmsson B.J., Aschard H., Blum M.G.B. (2019). Making the Most of Clumping and Thresholding for Polygenic Scores. Am. J. Hum. Genet..

[36] Cao C., Zhang S., Wang J., Tian M., Ji X., Huang D., Yang S., Gu N. (2024). PGS-Depot: A comprehensive resource for polygenic scores constructed by summary statistics based methods. Nucleic Acids Res..

[37] Privé F., Aschard H., Ziyatdinov A., Blum M.G.B. (2018). Efficient analysis of large-scale genome-wide data with two R packages: Bigstatsr and bigsnpr. Bioinformatics.

[38] Zuo F., Wang Y., Xu X., Ding R., Tang W., Sun Y., Wang X., Zhang Y., Wu J., Xie Y. (2024). CCDC92 deficiency ameliorates podocyte lipotoxicity in diabetic kidney disease. Metab. Clin. Exp..

[39] Tucker E.J., Wanschers B.F., Szklarczyk R., Mountford H.S., Wijeyeratne X.W., van den Brand M.A., Leenders A.M., Rodenburg R.J., Reljić B., Compton A.G. (2013). Mutations in the UQCC1-interacting protein, UQCC2, cause human complex III deficiency associated with perturbed cytochrome b protein expression. PLoS Genet..

[40] Winkler T.W., Justice A.E., Graff M., Barata L., Feitosa M.F., Chu S., Czajkowski J., Esko T., Fall T., Kilpeläinen T.O. (2015). The Influence of Age and Sex on Genetic Associations with Adult Body Size and Shape: A Large-Scale Genome-Wide Interaction Study. PLoS Genet..

[41] Rask-Andersen M., Karlsson T., Ek W.E., Johansson Å. (2019). Genome-wide association study of body fat distribution identifies adiposity loci and sex-specific genetic effects. Nat. Commun..

[42] Yoon K.H., Lee J.H., Kim J.W., Cho J.H., Choi Y.H., Ko S.H., Zimmet P., Son H.Y. (2006). Epidemic obesity and type 2 diabetes in Asia. Lancet (Lond. Engl.).

[43] Lotta L.A., Wittemans L.B.L., Zuber V., Stewart I.D., Sharp S.J., Luan J., Day F.R., Li C., Bowker N., Cai L. (2018). Association of Genetic Variants Related to Gluteofemoral vs Abdominal Fat Distribution With Type 2 Diabetes, Coronary Disease, and Cardiovascular Risk Factors. Jama.

[44] Emdin C.A., Khera A.V., Natarajan P., Klarin D., Zekavat S.M., Hsiao A.J., Kathiresan S. (2017). Genetic Association of Waist-to-Hip Ratio With Cardiometabolic Traits, Type 2 Diabetes, and Coronary Heart Disease. Jama.

[45] Karpe F., Pinnick K.E. (2015). Biology of upper-body and lower-body adipose tissue--link to whole-body phenotypes. Nat. reviews. Endocrinol..

[46] Tramunt B., Smati S., Grandgeorge N., Lenfant F., Arnal J.F., Montagner A., Gourdy P. (2020). Sex differences in metabolic regulation and diabetes susceptibility. Diabetologia.

[47] Mauvais-Jarvis F. (2015). Sex differences in metabolic homeostasis, diabetes, and obesity. Biol. Sex. Differ..

[48] Yusuf S., Hawken S., Ounpuu S., Bautista L., Franzosi M.G., Commerford P., Lang C.C., Rumboldt Z., Onen C.L., Lisheng L. (2005). Obesity and the risk of myocardial infarction in 27,000 participants from 52 countries: A case-control study. Lancet (Lond. Engl.).

[49] Klarin D., Lynch J., Aragam K., Chaffin M., Assimes T.L., Huang J., Lee K.M., Shao Q., Huffman J.E., Natarajan P. (2019). Genome-wide association study of peripheral artery disease in the Million Veteran Program. Nat. Med..

[50] Ngo D.T., Farb M.G., Kikuchi R., Karki S., Tiwari S., Bigornia S.J., Bates D.O., LaValley M.P., Hamburg N.M., Vita J.A. (2014). Antiangiogenic actions of vascular endothelial growth factor-A165b, an inhibitory isoform of vascular endothelial growth factor-A, in human obesity. Circulation.

[51] Kikuchi R., Nakamura K., MacLauchlan S., Ngo D.T., Shimizu I., Fuster J.J., Katanasaka Y., Yoshida S., Qiu Y., Yamaguchi T.P. (2014). An antiangiogenic isoform of VEGF-A contributes to impaired vascularization in peripheral artery disease. Nat. Med..

[52] He J., Quintana M.T., Sullivan J., Parry T.L., Grevengoed J.T., Schisler J.C., Hill J.A., Yates C.C., Mapanga R.F., Essop M.F. (2015). MuRF2 regulates PPARγ1 activity to protect against diabetic cardiomyopathy and enhance weight gain induced by a high fat diet. Cardiovasc. Diabetol..

[53] Ntambi J.M., Liu X., Burhans M.S., ALjohani A., Selen E.S., Kalyesubula M., Assadi-Porter F. (2023). Hepatic oleate regulates one-carbon metabolism during high carbohydrate feeding. Biochem. Biophys. Res. Commun..

[54] Breyer M.K., Spruit M.A., Hanson C.K., Franssen F.M., Vanfleteren L.E., Groenen M.T., Bruijnzeel P.L., Wouters E.F., Rutten E.P. (2014). Prevalence of metabolic syndrome in COPD patients and its consequences. PLoS ONE.

[55] Chen Z., Salam M.T., Alderete T.L., Habre R., Bastain T.M., Berhane K., Gilliland F.D. (2017). Effects of Childhood Asthma on the Development of Obesity among School-aged Children. Am. J. Respir. Crit. Care Med..

[56] Camp P.G., Coxson H.O., Levy R.D., Pillai S.G., Anderson W., Vestbo J., Kennedy S.M., Silverman E.K., Lomas D.A., Paré P.D. (2009). Sex differences in emphysema and airway disease in smokers. Chest.

[57] Santos F.M.D., Viana K.P., Saturnino L.T., Lazaridis E., Gazzotti M.R., Stelmach R., Soares C. (2018). Trend of self-reported asthma prevalence in Brazil from 2003 to 2013 in adults and factors associated with prevalence. J. Bras. Pneumol. Publicacao Soc. Bras. Pneumol. E Tisilogia.

[58] Sideleva O., Suratt B.T., Black K.E., Tharp W.G., Pratley R.E., Forgione P., Dienz O., Irvin C.G., Dixon A.E. (2012). Obesity and asthma: An inflammatory disease of adipose tissue not the airway. Am. J. Respir. Crit. Care Med..

[59] Reyes C., Leyland K.M., Peat G., Cooper C., Arden N.K., Prieto-Alhambra D. (2016). Association Between Overweight and Obesity and Risk of Clinically Diagnosed Knee, Hip, and Hand Osteoarthritis: A Population-Based Cohort Study. Arthritis Rheumatol..

[60] Collins J.E., Deshpande B.R., Katz J.N., Losina E. (2016). Race- and Sex-Specific Incidence Rates and Predictors of Total Knee Arthroplasty: Seven-Year Data From the Osteoarthritis Initiative. Arthritis Care Res..

[61] Lou L., Ye X., Xu P., Wang J., Xu Y., Jin K., Ye J. (2018). Association of Sex With the Global Burden of Cataract. JAMA Ophthalmol..

[62] Zetterberg M., Celojevic D. (2015). Gender and cataract--the role of estrogen. Curr. Eye Res..

[63] Nilsson M., Johnsen R., Ye W., Hveem K., Lagergren J. (2003). Obesity and estrogen as risk factors for gastroesophageal reflux symptoms. Jama.

[64] Kubo A., Cook M.B., Shaheen N.J., Vaughan T.L., Whiteman D.C., Murray L., Corley D.A. (2013). Sex-specific associations between body mass index, waist circumference and the risk of Barrett’s oesophagus: A pooled analysis from the international BEACON consortium. Gut.

[65] Alansari Sr A.H., Almalawi A.M., Alghamdi A., Alghamdi M.S., Hazazi H.A., Aljabri A.A., Alsulami R.A., Alkhoshi A.M., Khinaifis F. (2023). Body Mass Index Within Multifactor Predictors of Ventral Hernia Recurrence: A Retrospective Cohort Study. Cureus.

[66] Yoo S., Sung M.W., Kim H. (2020). CT-defined visceral adipose tissue thresholds for identifying metabolic complications: A cross-sectional study in the United Arab Emirates. BMJ Open.

[67] Fox C.S., Massaro J.M., Hoffmann U., Pou K.M., Maurovich-Horvat P., Liu C.Y., Vasan R.S., Murabito J.M., Meigs J.B., Cupples L.A. (2007). Abdominal visceral and subcutaneous adipose tissue compartments: Association with metabolic risk factors in the Framingham Heart Study. Circulation.

[68] Furman D., Campisi J., Verdin E., Carrera-Bastos P., Targ S., Franceschi C., Ferrucci L., Gilroy D.W., Fasano A., Miller G.W. (2019). Chronic inflammation in the etiology of disease across the life span. Nat. Med..

[69] Ouchi N., Parker J.L., Lugus J.J., Walsh K. (2011). Adipokines in inflammation and metabolic disease. Nat. Rev. Immunol..

[70] Lonardo A., Nascimbeni F., Ballestri S., Fairweather D., Win S., Than T.A., Abdelmalek M.F., Suzuki A. (2019). Sex Differences in Nonalcoholic Fatty Liver Disease: State of the Art and Identification of Research Gaps. Hepatology.

[71] Klop B., Elte J.W., Cabezas M.C. (2013). Dyslipidemia in obesity: Mechanisms and potential targets. Nutrients.

[72] Liu Y., Fan Y., Liu Q., Liu K., Chen F., Tang X., Li G., Hu D., Song G. (2020). Sex-specific association of serum uric acid dynamics with the incidence of metabolic syndrome in a health check-up Chinese population: A prospective cohort study. BMJ Open.

[73] Torkamani A., Wineinger N.E., Topol E.J. (2018). The personal and clinical utility of polygenic risk scores. Nat. Rev. Genet..

[74] Lewis A.C.F., Green R.C., Vassy J.L. (2021). Polygenic risk scores in the clinic: Translating risk into action. HGG Adv..

[75] Chatterjee N., Shi J., García-Closas M. (2016). Developing and evaluating polygenic risk prediction models for stratified disease prevention. Nat. Rev. Genet..

