# Supplementary material for: Investigating the Sexual Dimorphism of Waist-to-Hip Ratio and Its Associations with Complex Traits
Source: Genes (Basel). 2025 Jun 16;16(6):711. doi: 10.3390/genes16060711 (PMC12193181; doi:10.3390/genes16060711)
Supplement: Supplementary file 1 [file genes-16-00711-s001.zip › Supplementary Figures.pdf]

Supplementary Figures

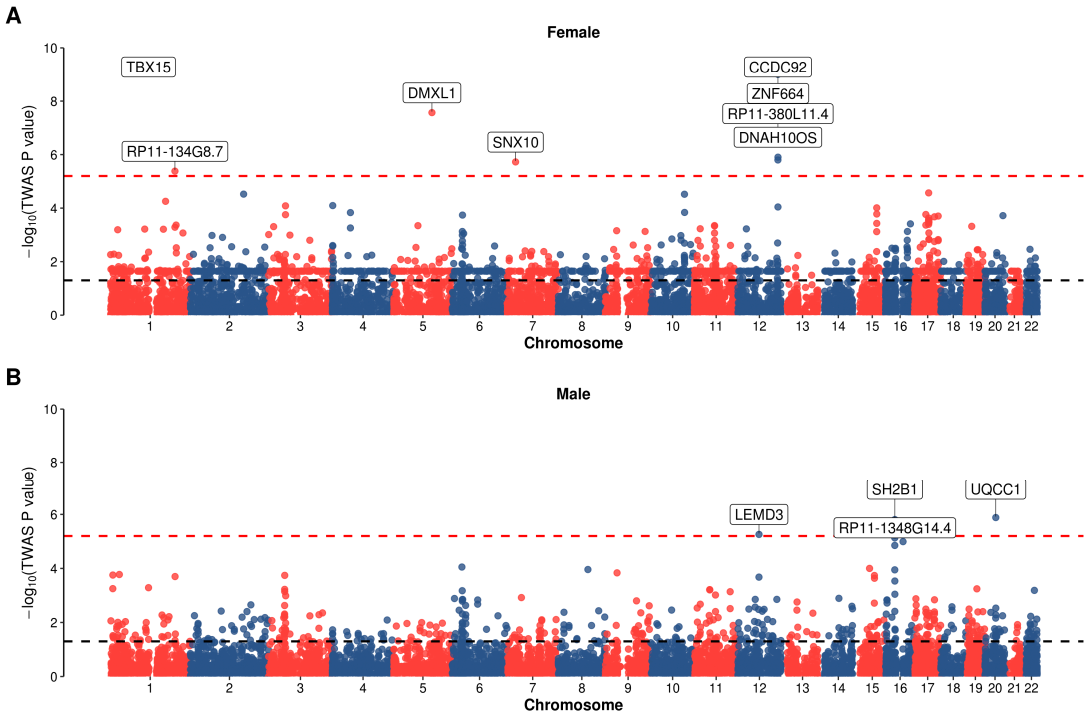

Figure S1 **TWAS results of WHR in adipose visceral omentum tissue. (A)** TWAS results of WHR in female set; **(B)** TWAS results of WHR in male set. Each dot represents a gene. The solid red horizontal line is marked at the Bonferroni threshold of significance for multiple testing, and the black dotted horizontal line represents the $P < 0.05$.

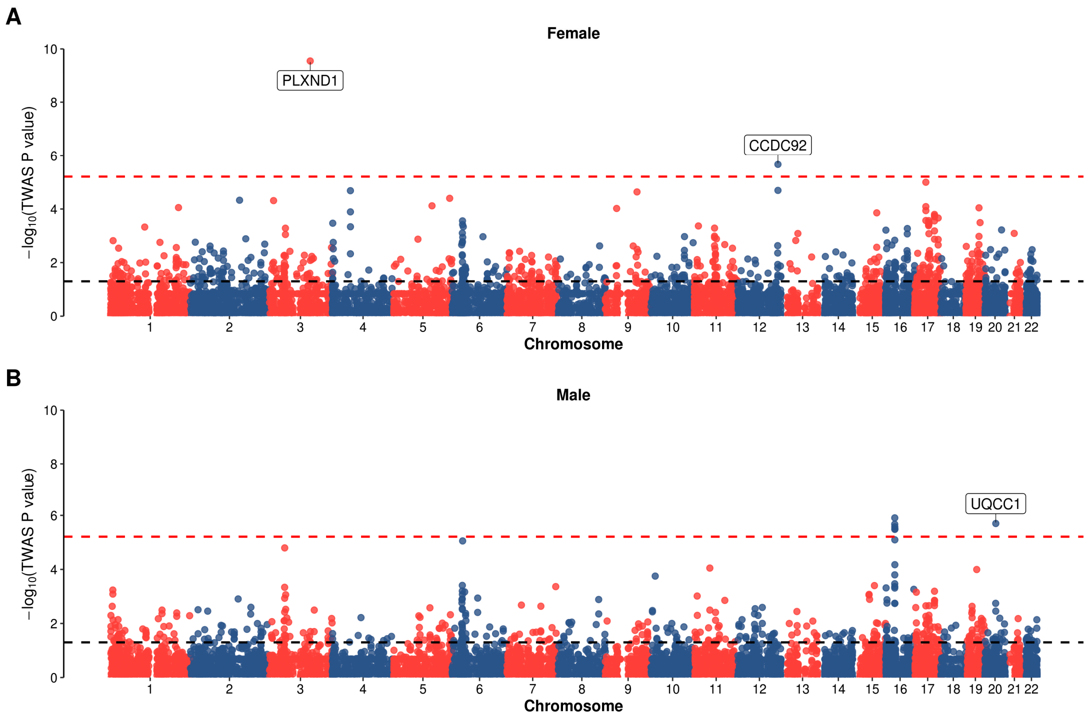

Figure S2 **TWAS results of WHR in whole blood tissue. (A)** TWAS results of WHR in female set; **(B)** TWAS results of WHR in male set. Each dot represents a gene. The solid red horizontal line is marked at the Bonferroni threshold of significance for multiple testing, and the black dotted horizontal line represents the $P < 0.05$.
